# The true history of the Hueter-Volkmann law

**DOI:** 10.1007/s00264-024-06254-w

**Published:** 2024-07-31

**Authors:** Jan Bartoníček, Ondřej Naňka

**Affiliations:** 1 Department of Orthopedics, First Faculty of Medicine, Charles University and the Central Military Hospital, U Vojenské nemocnice 1200, Prague 6, Czech Republic; 2 Institute of Anatomy, First Faculty of Medicine, Charles University, U Nemocnice 3, 128 00 Prague 2, Czech Republic

**Keywords:** Hueter-Volkmann law, Wollf law, History, Bone growth, Physis

## Abstract

**Introduction:**

The Hueter-Volkmann law (HVL) of the response of growth plate to compression load is a basic concept in orthopaedics. However, little is known about the origin of HVL and its history.

**Materials and methods:**

A literature search was performed in original publications and historical sources.

**Results:**

An analysis of all Volkmann´s and Hueter´s texts has shown that none of their publications was based on experiments, but on the data in the literature and their own clinical observations. They did not deal at all with the effect of pressure on the growth plate and mentioned this structure only marginally. The authors coined the opinion that increased pressure retards and decreased pressure accelerates bone growth. Julius Wolff criticized the HVL and concentrated all his arguments in the book “The law of bone remodeling”. According to him, increased pressure leads to bone formation, decreased pressure to its resorption. The Wolff-Volkmann dispute was addressed in the German literature by a number of authors. Walther Müller in his monograph “The normal and pathological physiology of the bone” criticized Wolff for his concept of interstitial bone growth. In Müller´s view, HVL applies to the growing bone and Wolff confuses growth with hypertrophy of the mature bone.

**Conclusion:**

The circumstances of the emergence of HVL are inaccurately and incompletely described in the current literature, as they are mostly taken from secondary sources. HVL, as it is presented today, is not the original formulation, but the result of a long historical evolution.

## Introduction

The Hueter-Volkmann law (HVL) of the response of growth plate to compression load is a basic concept in paediatric orthopaedics and traumatology, which has been discussed in the osteological and orthopaedic literature for more than 150 years since its first formulation [1–10]. Its current version states in a simplified way that “*increased compression of the physis reduces the growth rate and*, vice versa,* reduced compression accelerates growth in length”* [2, 7, 9]. However, little is known about the circumstances of the origin of HVL, its authors or its history. Only one, very brief study can be found in the literature, which is far from covering the whole issue and, moreover, contains a number of inaccuracies [4]. Other articles mention HVL only marginally and very inaccurately, including erroneous, or incomplete, original citations [2–4, 7]. However, HVL cannot be properly grasped without the knowledge of the context of the time of its origin.

## Osteology in mid-19^th^ century

The beginning of the second half of 19th century was a remarkable period from the viewpoint of the study of bone growth. At that time, the so-called Hunter-Flourens´ theory [10–12] was gradually spreading, stating that a long bone grows in length only at its epiphyseal ends and its final shape is the result of apposition and resorption processes which may occur simultaneously on the periosteal and intramedullary surfaces of the bone [13, 14]. Several years before (1836), *“an ossifying zone”* (growth plate) between diaphysis and epiphysis was described [15], and following studies brought new findings about its importance for longitudinal growth of long bones [16–20]. All this was occurring at the time when biological sciences were considerably influenced by the cell theory. The interest in bone growth was further intensified by the study of rachitis and skeletal syphilis [21, 22].

New findings also posed new questions, one of which concerned the factors influencing the growth of bones and their resulting shape and structure. A lively discussion on this topic developed in the 1860s. It was at that time when two prominent German surgeons, Richard von Volkmann and Carl Hueter, entered the scene and began, independently of each other, to address this issue.

## HVL “authors”

***Richard von Volkmann (1830–1887)*** came from a famous medical family. His father Alfred Volkmann (1801–1877) became professor of physiology and anatomy at the University of Halle, and his name was used as an eponym for the transverse (Volkmann´s) canals in the Haversian system of the cortical bone.

His son Richard enrolled in the medical school at the University of Halle. Later he continued his studies in Giessen, Heidelberg, and finished them in Berlin. There he met another extraordinary personality of the German and world surgery, Konrad Bernhard von Langenbeck (1810–1887). After passing state exams in 1855, Volkmann accepted a post at the Surgical Clinic in Halle, led by Professor Ernst Blasius (1802–1875), and was appointed extraordinary professor in 1863, then full professor in 1867. In the same year he became head of the Surgical Clinic. In 1870, he founded the journal “*Sammlung klinischer Vorträge*“ (Collection of Clinical Reports). Due to his poor health, he refused to succeed Langenbeck in Berlin in 1882. His international reputation was so high that even Pope Pius IX consulted him in 1885 about his foot ailment. Richard von Volkmann died at the age of only 59 years from spinal cord malacia [23].

Volkmann was a surgeon of extraordinary importance. He was co-founder of the German Society for Surgery (1872). In 1873, he introduced the Lister method of antisepsis into Germany. He was extensively involved in bone and joint surgery, e.g., hip or knee osteotomies, muscular torticollis, bone tumors, club-foot, poliomyelitis, infectious bone and joint diseases, patellar fractures, etc. [24]. His description of ischaemic contracture in paediatric supracondylar fracture, published in 1881, became world famous [25] and eponymous. The term “Volkmann’s triangle, or fragment” had been used in the literature for many decades, although wrongly, to refer to the avulsed posterior malleolus in ankle fracture dislocations [26].

***Carl Hueter (1838–1882)*** was a pupil of Rudolf Virchow, and later of Konrad von Langenbeck. After completion of his medical studies in 1858, he continued his education in Berlin, Vienna, England and Paris. He started his independent career in 1868 in Rostock, and in 1877 he moved to Greifswald. The cause of his untimely death was morbid obesity.

Hueter focused on joint diseases and summarized his findings in the book “*Klinik der Gelenkkrankheiten mit Einschluss der Orthopädie“*, published in 1870. He was a co-founder of the prominent journal “*Deutsche Zeitschrift für Chirurgie“.* Hueter was also the promoter of the term “hallux valgus“, and his name is associated with resection of the metatarsal head for infection of this deformity [4].

## The first formulations of HVL

Three articles, published in the Virchow archives in 1862 and 1863 [27–29] are referred to as the beginnings of the HVL formulation. Unfortunately, they are all written in a very complicated and lengthy manner and it is not easy to interpret their content accurately.

**Fig. 1:**
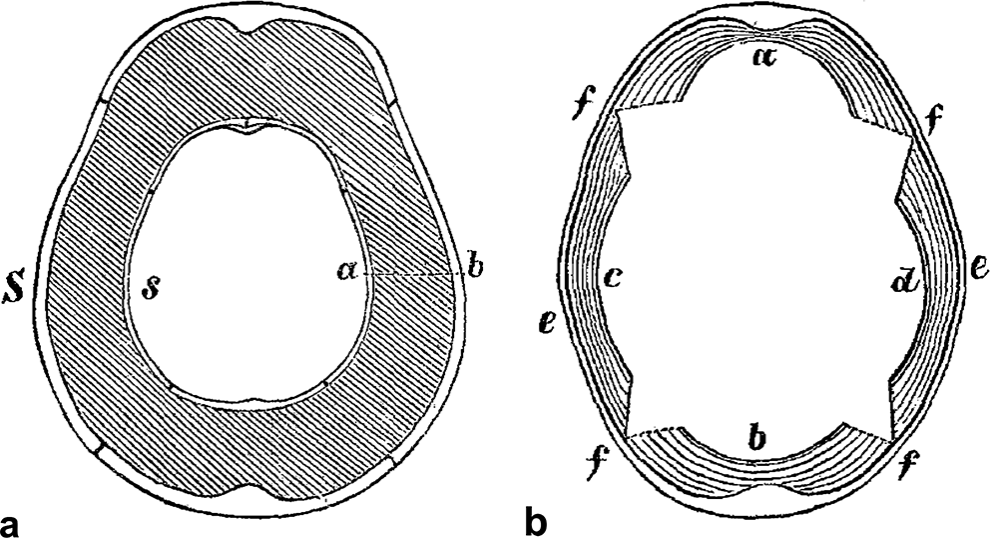
The growth of the human skull, after Volkmann [26]. **a** – change in the skull shape: *S* – curvature of an adult skull, *s* – curvature of a child´s skull, *a-b* – shift of the double contour of parietal bone during growth; **b** – change in skull curvature: *a*,* b*,*c*,* d* – curvature of individual bones forming the calvaria of a child´s skull, *e* – the double contour of an adult, *f*,* ff*,* f* – cross-sections of skull sutures, which are the regions of the minimal periosteal apposition of the bone

**Fig. 2:**
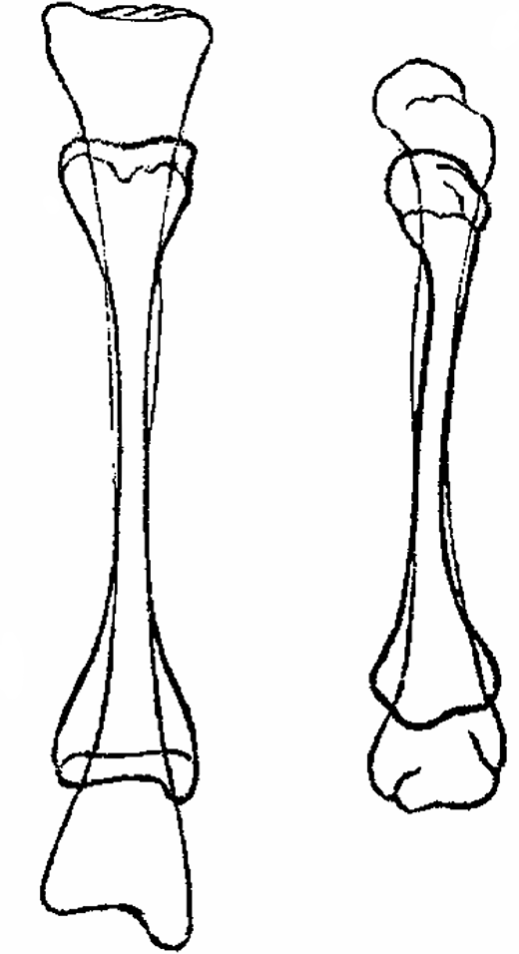
Interstitial bone growth of long bone after Volkmann [26]

Volkmann was the first to publish his article (1862) [27] focused on bone growth, which, however, was based only on an analysis of the literature and his own clinical observations, rather than on experiments. Most authors who dealt with HVL, reduced Volkmann´s article to a mere analysis of the growth of the human skull [4, 7]. However, Volkmann addressed more issues in his study and his opinions may be summarized as follows:

- Growth of bone and maintenance of its shape must be controlled by certain laws.
- One of the laws states that during growth, bone shape changes mostly by periosteal and by simultaneous intramedullary apposition.
- A child’s skull has a sharper curvature than an adult skull. A child´s skull grows in such a way that “…*there is never any overstressing of the bone… the periosteum adds new layers*,* while the increasing pressure from the inside on the previously formed bone leads to its atrophy*”. Thereby, the internal processes taking place in the bone also influence its external shape (Fig. 1).

- The change in the shape of the bone occurs for various reasons. An example may be genu valgum and genu varum, when the physiological axis can be restored by application of plaster fixation and splints for 6 months. The bone shape may be changed also due to bending, or shifting, of the whole articular surface, e.g. in osteoarthritis, or bending of ribs in scoliosis. Other causes include for instance inactivity (senility, palsy etc.).
- Bone elasticity and calcium metabolism are tools to change gradually the bone shape.
- A bone grows interstitially and, as a result, it may enlarge or shrink (Fig. 2).

- Fractures or necroses may result in overgrowth of the extremity, caused by inflammation stimulating a higher production of cells in the nearby epiphyseal cartilage. Conversely, certain inflammations, suppurations, or synostoses cause shortening of the bone.

**Fig. 3:**
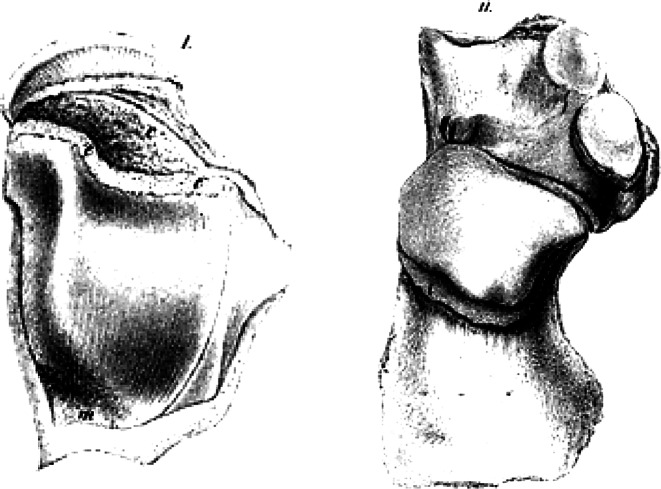
Original drawings of talus and calcaneus used by Hueter to study bone growth in the region of the subtalar joint [27]

Another study on this topic was published by Hueter, in 1862 [28], a few months after Volkmann, the second part of which was published as late as in 1863 [29], and is referred to in the literature only rarely [4]. In the first part of his study, Hueter dealt with articulation between the tibia and talus, and also with analysis of the subtalar joint in both children and adults (Fig. 3). Based on his observations, he deduced that the shape of the articulating bones resulted primarily from a relatively higher growth rate of those parts of the bone that were exposed to a lower compression load. In the other part of the article, the author focused on knee and hip joints. He discussed in detail the shape of the articular surfaces and noticed that the femur grows much more at its distal end. He described the division of the common cartilaginous epiphysis of the proximal femur into two parts. Worth noting is the part where, referring to Duhamel [30], Hunter [11] and Flourens [12], he argued with Volkmann about interstitial bone growth (IBG). Hueter was very cautious about the theory of interstitial growth and claimed that further discussion was needed in this direction.

One year later (1864), Hueter published a study on mandibular growth in which he again discussed Volkmann’s views [31]. This article, however, is not mentioned in the HVL literature.

### Note

Mandibular growth fascinated many prominent anatomists and surgeons for many decades [11–14]. Unfortunately, it was not a suitable bone for studying the general principles of bone growth, as mandibular growth is complicated and could be most easily explained by IBG.

Another source, often referred to by authors dealing with HVL [4, 7], is Volkmann´s chapter *Die Krankheiten der Bewegungsorgane* in the monumental textbook of surgery “*Handbuch der allgemeinen und speziellen Chirurgie*“ published in 1865–1882 by Theodor Billroth (1829–1894) and František Piťha (1810–1875). However, individual authors differ on the year of publication, stating the years 1865 [7], 1869 [4] or 1882 [3]. We have discovered that 1865 is the year of the first edition [32] and 1882 of the second edition [33], although it is mentioned in neither book title. The year 1869 was obviously a mistake.

In both editions, Volkmann´s chapter is included in *Band II*,* Abteilung II.* Here, in Chap. 3*7 ”Bone hypertrophy and atrophy”*, the author briefly states on pages 350–351 that hypertrophic conditions may include *“abnormal lengthening of the bone due to absence of physiological compression load*,* an analogue of the much better known and much more common pressure atrophy*,* or pressure usuration: increased growth of the radius in length after its dislocation in the humeroradial joint…”* [32, 33].

On page 333, in both editions, Volkmann mentioned bone growth. He stated that it occurs in three ways. Responsible for longitudinal growth is the cartilaginous disc between the epiphysis and metaphysis. Cartilaginous cells proliferate on the epiphyseal side and turn into bone cells on the diaphyseal side. Bone growth in width is the function of the periosteum. Widening of the medullary canal occurs by endosteal resorption. On pages 692–700 of the same chapter, Volkmann focused on the effect of compressive load on the shape of articular surfaces: “*Anomalous difference in pressure results in asymmetric growth of the articular ends. Greater pressure retards the growth*,* lower pressure accelerates it“.*

No mention was made of IBG there, which contradicted the theory of interstitial growth that he had published in 1862 [27]. Volkmann defended the IBG theory again in 1870, stating that a bone grows from the physis (Epiphysenfuge) only minimally [34]:“…*I am convinced that the great long bones grow in length due to interstitial growth*,* and involvement of the growth cartilage comes into consideration only minimally.“.* Such a fundamental change in his views is hard to explain, perhaps only by the fact that in the official textbook of surgery he presented the majority opinion of that time, whereas in the two articles he presented his own standpoint.

A careful analysis of all Volkmann´s and Hueter´s texts has shown that none of their publications [27–29, 31–34] was based on experiments, but merely on the data in the literature and their own anatomical and clinical observations. With some exaggeration it may be said that both authors, and particularly Volkmann, used incompatible arguments. They did not deal at all with the effect of pressure on the growth plate and mentioned this structure only marginally. They differed on a number of aspects, such as interstitial growth, and shared only the opinion that ***increased pressure retards and decreased pressure accelerates bone growth***.

The statement by Willy et al. [7] concerning Volkmann saying that he also ‘‘*suggested alterations in the growth of long bones as a result of tension and compression on the epiphyseal plate.*.’’, is a deep historical error. The way HVL is presented today [2, 4, 7, 9] is the result of gradual distortion of the original facts passed on from one article to another, when most modern authors do not know the original texts.

## Dispute with Wolff

The German orthopedist, ***Julius Wolff (1836–1902)***, in 1868 [35], criticized the Hunter-Flourens´ theory, on the basis of his own experiments, the results of which differed from the findings of the quoted authors [11, 12]. Wolff, similar to Volkmann, was a keen supporter of IBG theory. He dealt with the structure of bone, especially the proximal femur, based on the ideas of the Swiss anatomist Herman von Meyer [36, 37]. Wolff considered pressure to be a critical factor stimulating growth of bones and influencing their external shape and internal architecture. He distinguished between three types of growth: (1) external growth according to Hunter and Flourens (periosteal and endosteal apposition and resorption), (2) internal expansive cellular and intercellular growth of a mature bone and (3) growth causing a change in bone internal architecture [38]. In his articles published later in the 1870s [39], he began to criticize also Volkmann´s opinions, quite rightly in a number of aspects. Hueter´s studies were mentioned by him only marginally.

This sparked a fierce debate in the German literature of the 1870s, mainly concerning the way that bone grew [13, 14]. The famous German pathologist ***Rudolf Virchow (1821–1902)*** [38] pointed out that growth without an increase in volume is not growth, and that growth should not be confused with a change in bone architecture. His pupil, another outstanding German pathologist ***Friedrich Rudolph Georg Wegner (1843–1917)***, criticized, in 1874 [14], Wolff´s experimental findings of 1868, as well as his concept of interstitial bone growth. Wegner supported the Hunter-Flourens´ theory on the basis of his own experiments and a detailed analysis of the literature, and claimed that interstitial growth cannot change the shape of a mature (adult) bone.

Wolff concentrated all his arguments against Volkmann in the book “*Das Gesetz der Transformation der Knochen“* (The law of bone remodeling) of 1891 [40]. Interestingly, there was almost no mention of IBG in the book. It is thus clearly evident that the original dispute from the 1870s about IBG, gradually escalated into a dispute about the functional adaptation of bone, i.e., about bone as an organ. The *“Volkmann-Hueter*,* or Compression Theory (Druck Theorie)”* was, in Wolff´s view, incompatible with the transformational law that he formulated. According to him, increased pressure leads to bone formation, decreased pressure to its resorption. Paradoxically, this long-standing criticism by Wolff of Volkmann-Hueter’s “Druck Theorie” did not lead to its elimination, but on the contrary made it famous.

In the following years, the Wolff-Volkmann dispute was addressed in the German literature by a number of authors. Among the most prominent was the German orthopedist ***Walther Müller (1888–1949)*** in his outstanding 1924 monograph “*Die normale und pathologische Physiologie des Knochens“* (The normal and pathological physiology of the bone) [41]. Müller was talking there about the Hueter-Volkmann theory, not the law. He criticized Wolff for his IBG concept. In Müller´s view, HVL applies to the growing bone and Wolff confuses growth with hypertrophy of the mature bone.

The German surgeon ***Ernst Bergmann*** presented, in 1931, an excellent overview of the then current knowledge of longitudinal bone growth [42]. In it he also commented on the so-called Hueter-Volkmann Lehre (theory), which, according to him, expresses the relationship between function and longitudinal bone growth … “*increased compression load hinders longitudinal growth*,* whereas reduced pressure leads to increased growth“.* However, according to Bergmann, this theory has certain limitations. He states that the main opponent of this theory is Wolff, who argues that increased pressure leads to bone building, decreased pressure to bone resorption. Bergmann himself was a proponent of appositional bone growth and carefully compared the arguments of both sides, noting that the problem is much more complex than it appears.

The Volkmann-Wolff dispute was presented in great detail by a Swiss orthopaedic surgeon ***Hans Debrunner*** in a comprehensive biographical study of Rudolf von Volkmann of 1932 [23].

After World War II (1950), the dispute Hueter-Volkmann versus Wolff was dealt with in detail by the German surgeon ***Heinz Gelbke*** [43] who already used the expression “the Hueter-Volkmann law” in the German literature, and in his views on the clash between the two concepts he agreed with Müller [41 ] and Bergmann [42].

## HVL in the English literature

It is surprising that until the end of World War II, HVL was rarely mentioned in the English orthopaedic and anatomical literature.

The turning point came in 1949, when two articles were published in the same issue of Journal of Bone and Joint Surgery (JBJS) [44, 45]. The first of them was published by Blount and Clarke [44], who focused on “*Control of bone growth by epiphyseal stapling*“. The authors briefly mentioned the first part of the Hueter´s study and the Volkmann´s chapter in the Billroth-Pitha textbook. In the other article, “*The mechanism of the structural changes in scoliosis*”, Arkin [45] states that “*The relationship between pressure and epiphyseal growth has been mentioned by … Hueter and Volkmann*“, referencing only the first Hueter´s study [27], and Volkmann´s chapter in the book of 1882 [33]. In the discussion at the end of the article, Risser pointed out “*the importance of the Hueter-Volkmann´s epiphyseal pressure rule*”. In 1951, Gelbke published an abridged version of his German experimental studies in JBJS [46]. In the introduction, he briefly mentioned Hueter´s and Volkmann´s concepts, as well as Wolff’s critical views on them. In 1956, Arkin and Katz [47] published a study on “*The effects of pressure on epiphyseal growth“*. The authors presented there, for the first time, the term Hueter-Volkman law in the English literature, claiming that in the American literature it refers to the effect of decreasing, or increasing, pressure on the bone growth rate.

### Experimental verification of HVL

**Fig. 4:**
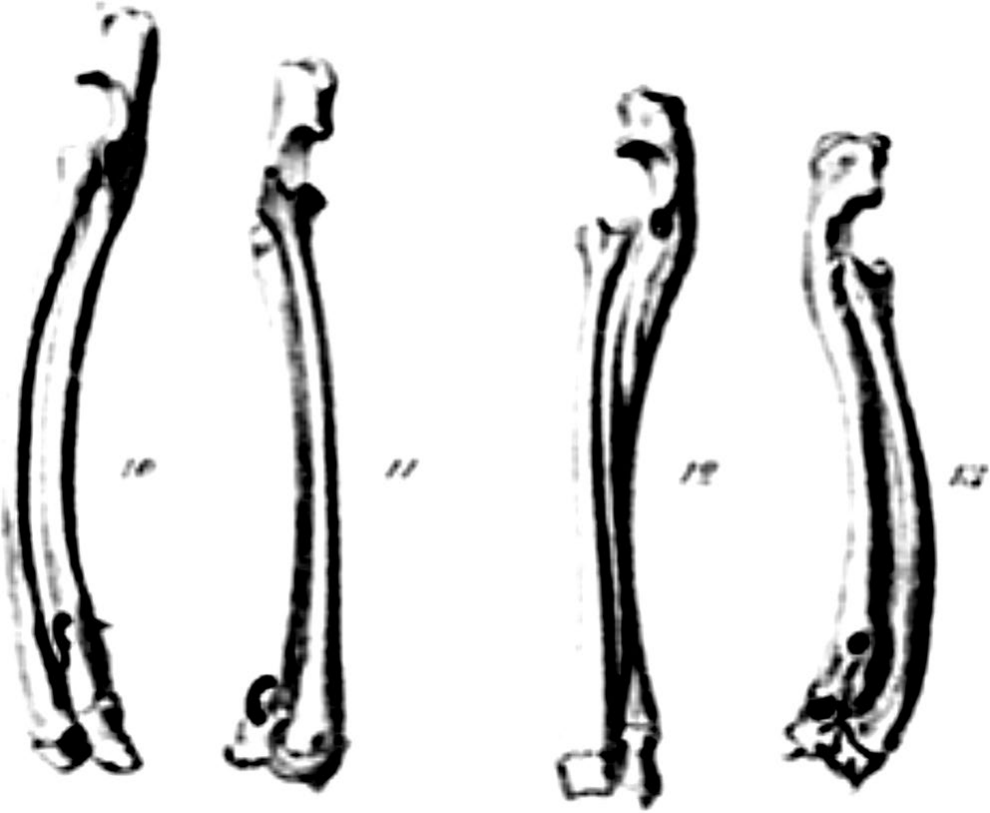
Original drawings of Wegner´s experiment on rabbits [13]. Blocking of growth of the distal ulnar physis by bridging metal staple. Deceleration of the growth of the distal ulna resulted in curvature of the distal radius

The first to verify experimentally the effect of pressure on bone growth was Wegner in 1874 [14]. In his experiments on rabbits, he bridged the distal physis of the ulna with a staple. Subsequently, he noted shortening of the ulna as compared to the other side, narrowing of the physis and microscopic changes in its structure (Fig. 4). Unfortunately, these experiments, as well as the whole Wegner´s study, despite their priority, have fallen into oblivion and are unknown in the current literature.

Wegner was followed by a number of authors. The best-known are the experimental studies by Müller [41], Haas [48, 49] and Gelbke [43], usually also conducted on rabbits, or dogs. All of them proved that increased pressure on physis results in slower growth. In clinical terms, the effect of pressure on growth retardation was demonstrated by Blount and Clarke [43] who blocked the distal physis of the femur by staples. In the following years, this method spread worldwide. This led to the recognition of the first part of the HVL, the problem was its second part. In evaluating his experiments, Gelbke [43, 46] stated that verifying the effect of tension on growth acceleration is a difficult task.

**Fig. 5:**
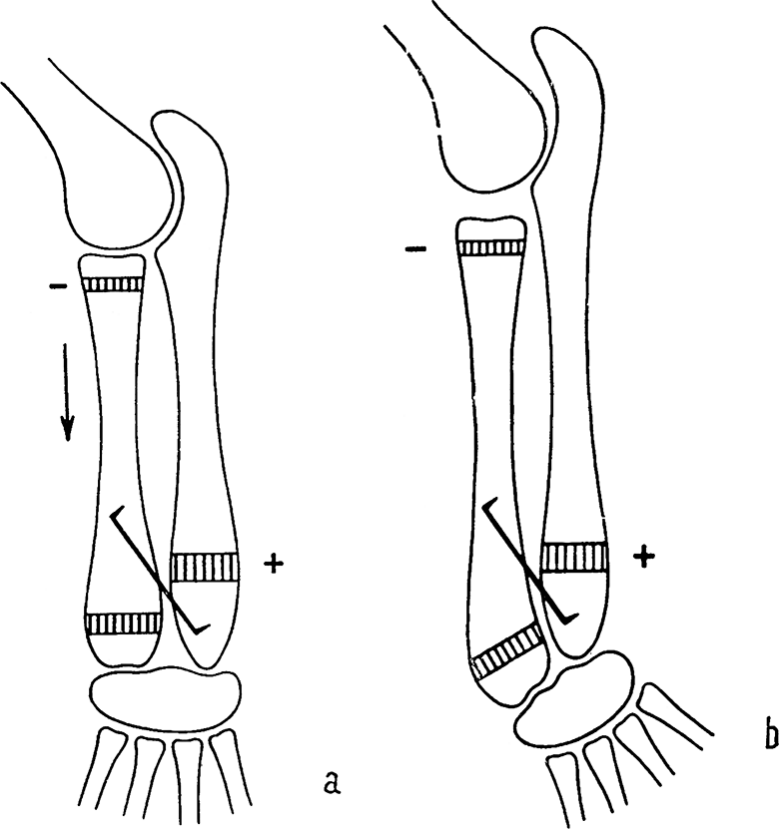
Original drawings of Heřt´s experiment reducing the pressure on the proximal physis of the radius of a rabbit [50]. **a** – fixation of the radial shaft together with the distal ulnar epiphysis by staple decreases pressure on the proximal radial physis (-) and increases pressure on the distal ulnar physis (+); arrow shows acceleration of the growth; **b** – the result of the experiment is acceleration of the growth of the radius and shortening of the ulna

**Fig. 6:**
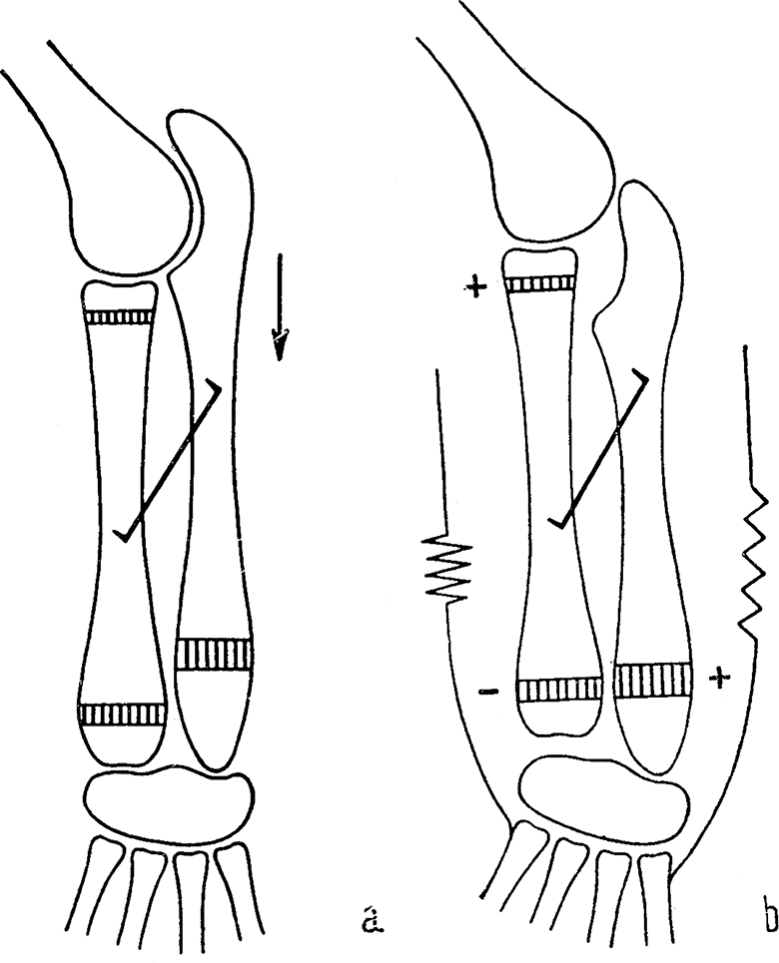
Original drawings of Heřt´s experiment increasing pressure on the proximal physis of the radius and the distal physis of the ulna in a rabbit [49]. **a** – fixation of the radial shaft together with the ulnar shaft prevents isolated shift of the radial shaft distally in relation to the ulna, caused by activity of the proximal radial physis. This shift is explained by the fact that longitudinal growth of the radius is ensured by two physes, whereas the ulna is a monoepiphyseal bone; **b** – the experiment has shown that the radial shaft pulls the ulna out of the elbow joint. The radius takes all the load in the elbow joint (+). At the distal end of the forearm, the radius grows more slowly than the ulna because of the absence of distal shift of the radius, which would compensate for the difference in activity of the distal physes of the radius and ulna. Initially, the ulna grows faster and takes over the load that is normally distributed between the two bones. Subsequently, the distal physis of the ulna is overloaded (+), whereas the pressure on the distal physis of the radius is reduced (-)

**Fig. 7:**
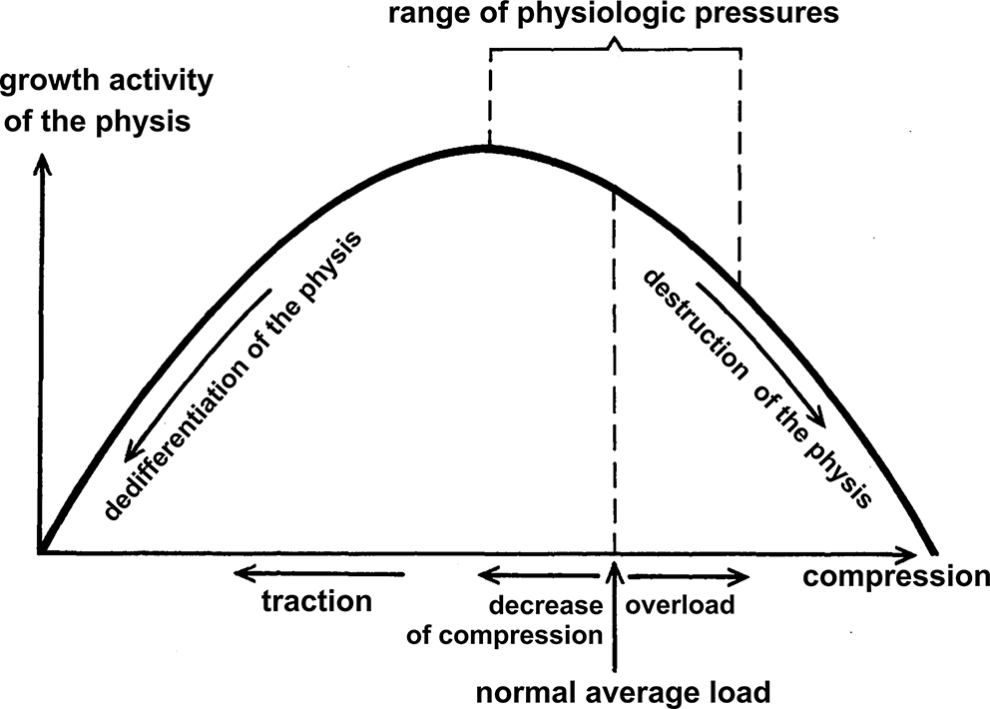
Heřt´s curve demonstrating the relationship between the load on the physis and its growth activity. Adapted after [50]

At the turn of the 1960, Hueter-Volkmann´s law was experimentally studied by the Czech anatomist ***Jiří Heřt (1928–2014)***. Through a series of elegant experiments on rabbits (Figs. 5 and 6), he confirmed its validity and refined its formulation. He summarized the results of his studies in 1964, i.e., 100 years after the first “HVL formulations” in a monograph “*Regulation of growth of long bones in length*“ published in Czech, but with an extensive English summary [50]. This study has a number of important priorities. Heřt later further specified his observations in other articles [51] where he stated that: *“HVL is valid only within a certain physiological range. When the pressure increases above this range*,* the physis gets damaged and disappears. In the absence of compression load*,* and particularly in tension*,* there occurs dedifferentiation of the physis followed by growth arrest“* (Fig. 7).

Heřt´s opinions have been cited and accepted in the English literature [2, 5]. His contribution was pointed out also by Mehlman et al. [4] in their historical study of 1997, dealing with Hueter-Volkmann law.

## Conclusion

The circumstances of the emergence of HVL are inaccurately and incompletely described in the current literature, as they are mostly taken from secondary sources. Few authors dealing with this topic have taken the trouble to wade through the difficult text of the original articles, not only by Volkmann and Huether, but also by their then advocates and critics. HVL, as it is presented today, is not the original formulation, but the result of a long historical evolution, as it is hard to imagine that Volkmann, a proponent of interstitial bone growth, would have formulated a law concerning the growth cartilage.

## Data Availability

This is a historical study based on original articles and books, as indicated in the References.
